# Molecular and agro-morphological characterization of new barley genotypes in arid environments

**DOI:** 10.1186/s12896-024-00861-6

**Published:** 2024-06-11

**Authors:** Adel A. Elshafei, Eid I. Ibrahim, Kamal F. Abdellatif, Abd El-Azeem K. Salem, Khaled A. Moustafa, Abdullah A. Al-Doss, Hussein M. Migdadi, Amal M. Hussien, Walid Soufan, Taha Abd El Rahman, Samah M. Eldemery

**Affiliations:** 1 Genetics and Cytology Department, Genetic Engineering and Biotechnology Research Institute, National Research Center, Dokki, Giza, 12622 Egypt; 2 Plant Production Department, College of Food and Agriculture Sciences, King Saud University, P.O. Box 2460, Riyadh, 11451 Saudi Arabia; 3 Plant Biotechnology Department, Genetic Engineering & Biotechnology Research Institute (GEBRI), University of Sadat City, Sadat, Egypt; 4 Field Crops Research Department, Agricultural and Biological Research Institute, National Research Center, Dokki, Giza, 12622 Egypt; 5 Barley Research Department, Field Crops Research Institute, Agricultural Research Center, Giza, 12619 Egypt; 6 Genetic Resources Research Department, Field Crops Research Institute, Agricultural Research Center, Giza, 12619 Egypt; 7 Département de Biologie, Faculté des Sciences, Université de Sherbrooke, Sherbrooke, QC J1K 2R1 Canada; 8 Molecular Biology Department, Genetic Engineering & Biotechnology Research Institute (GEBRI), University of Sadat City, Sadat, Egypt

**Keywords:** Barley, Biodiversity, Molecular markers, STRUCTURE, PCoA

## Abstract

**Background:**

Genetic diversity, population structure, agro-morphological traits, and molecular characteristics, are crucial for either preserving genetic resources or developing new cultivars. Due to climate change, water availability for agricultural use is progressively diminishing. This study used 100 molecular markers (25 TRAP, 22 SRAP, 23 ISTR, and 30 SSR). Additionally, 15 morphological characteristics were utilized to evaluate the optimal agronomic traits of 12 different barley genotypes under arid conditions.

**Results:**

Substantial variations, ranging from significant to highly significant, were observed in the 15 agromorphological parameters evaluated among the 12 genotypes. The KSU-B101 barley genotype demonstrated superior performance in five specific traits: spike number per plant, 100-grain weight, spike number per square meter, harvest index, and grain yield. These results indicate its potential for achieving high yields in arid regions.

The Sahrawy barley genotype exhibited the highest values across five parameters, namely leaf area, spike weight per plant, spike length, spike weight per square meter, and biological yield, making it a promising candidate for animal feed. The KSU-B105 genotype exhibited early maturity and a high grain count per spike, which reflects its early maturity and ability to produce a high number of grains per spike. This suggests its suitability for both animal feed and human food in arid areas. Based on marker data, the molecular study found that the similarity coefficients between the barley genotypes ranged from 0.48 to 0.80, with an average of 0.64. The dendrogram constructed from these data revealed three distinct clusters with a similarity coefficient of 0.80. Notably, the correlation between the dendrogram and its similarity matrix was high (0.903), indicating its accuracy in depicting the genetic relationships. The combined analysis revealed a moderate correlation between the morphological and molecular analysis, suggesting alignment between the two characterization methods.

**Conclusions:**

The morphological and molecular analyses of the 12 barley genotypes in this study effectively revealed the varied genetic characteristics of their agro-performance in arid conditions. KSU-B101, Sahrawy, and KSU-B105 have emerged as promising candidates for different agricultural applications in arid regions. Further research on these genotypes could reveal their full potential for breeding programs.

**Supplementary Information:**

The online version contains supplementary material available at 10.1186/s12896-024-00861-6.

## Background

Barley (*Hordeum vulgare* L.) is a significant cereal crop that ranks fourth in the most important cereal crop. According to FAO 2022, barley is the fifth most extensively cultivated cereal worldwide, covering approximately 47 million hectares [1]. In addition, it has been considered a model for ecological adaptation, ranging from the nearly subarctic to the subtropical regions. In the Middle East and North Africa, only 10% of barley is allocated for human food, while the remaining portion is used for animal feed and beer business [2]. Barley varieties are classified based on various variables, including the season in which they are grown (spring or winter), the number of rows of kernels per spike (two rows or six rows), and the existence of hull around the grain (has hulls or hullless) [3]. Considering the significance of barley in multiple industries, it is crucial to enhance barley yields. Barley plants can thrive in severe environmental conditions, such as high soil salinity, high temperature, and drought [4, 5]. To achieve high yield potential under a wide range of environmental challenges, barley breeders must develop new barley cultivars that can flourish under various environmental stresses [6].

Plant breeders look for genetic variations and particular traits that have the potential to improve and adapt crops. The origins of landraces and crop wild relatives exhibit the most exceptional genetic variation [7, 8]. Genetic divergence enables the identification of relevant genotypes for use as parental lines in planned crossings, as well as the separation of desirable progenies for selection [9]. In order for enhanced cultivars to arise, a genetic variation of economic traits must be present in the genetic pool. Genetic variation may decrease over time due to selective breeding and climatic change, leading to cumulative deprivation of genetic variability among crop species [10, 11].

Molecular markers have been used in several studies on barley to measure genetic variation in various germplasm collections. However, the majority of previous studies have mainly focused on cultivar collections or combinations of cultivars and landraces [12]. Molecular markers have been used in phylogenetic and species evolution research to enhance our understanding of the geographic distribution and extent of genetic variation within and across species [13]. Initially, high-throughput genotyping methods utilized many molecular markers such as SSR, SRAP, TRAP, ISSR, RAPD, and ISTR to detect numerous genetic variations in a single test, enabling the simultaneous identification of hundreds to thousands of polymorphisms in a single test [14–16]. SSR markers have been extensively utilized in various crops due to their multi-allelic nature [17, 18].

This study aimed to demonstrate the genetic variation of barley genotypes under arid conditions. Fifteen significant agromorphological traits, including growth, physiological, and yield component traits, were used to characterize 12 novel barley genotypes agromorphologically. Moreover, molecular markers such as ISTR, SRAP, TRAP, and SSR were used to examine the genetic variation of the barley genotypes.

## Materials and methods

### Plant materials

For this study, a total of 12 different barley genotypes were used. These included five elite varieties (Giza124, Giza121, Giza126, Sahrawy, and Giza123) from the barley breeding program at the Barley Research Department (BRD), Field Crops Research Institute (FCRI), Agriculture Research Center (ARC), Egypt. Additionally, the study suggested cultivars (Gusto) and a local Saudi line (Asser) in addition to five advanced lines (KSU-B101, KSU-B102, KSU-B103, KSU-B104, and KSU-B105) selected from the barley breeding programs during the 1990 growing season at the Dirab Agriculture Research Station, College of Food and Agriculture Sciences, King Saud University, Riyadh, KSA. A diallel cross was conducted using five parents (Gusto, C.C. 89, Giza 121, Giza 123, and Giza 124), with reciprocals excluded. During the 1991 growing season, the F_2_ generation of each cross was acquired. On January 1, 1992, in the early winter, the five parents and their 10 F_2_ segregating generations were seeded (Tables S1 and S2) [19]. Advanced promising lines were obtained from barley breeding programs for dual purposes (i.e., high grain yield as well as biological yield for animal feed) [20]. The selected genotypes were obtained from ICARDA, KSU, and ARC, Egypt (Table 1).

**Table 1:** Barley genotypes used in the present study and their pedigrees

No.	Genotypes	Type	Pedigree	Origin	Notes
1	KSU B101	Six rowed	G 121 X Gustoe	Saudi Arabia	Spring Habit
2	KSU B102	Six rowed	G 123 X Gustoe	Saudi Arabia	Salt tolerant
3	Giza 124	Six rowed	Giza 117/Bahteem 52//Giza 118/FAO 86	Egypt	Heat tolerant
4	Gustoe	Six rowed	No data	USA	Spring Habit
5	KSU B103	Two rowed	C.C.89 X G 123	Saudi Arabia	Salt tolerant
6	Giza 121	Six rowed	Baladi 16 X Astel	Egypt	Spring Habit
7	KSU B104	Two rowed	C.C.89 X G 124	Saudi Arabia	Heat tolerant
8	Giza 126	Six rowed	BaladiBahteem/SD729-Por12762-BC	Egypt	Drought tolerant
9	Sahrawy	Two rowed	Baladi 16 X Gem	Egypt	-
10	KSU B105	Two rowed	Rihanna X Lignee	Saudi Arabia	-
11	Asser	Two rowed	Local Variety	Saudi Arabia	-
12	Giza 123	Six rowed	Giza 117/FAO 86	Egypt	Salt tolerant

## Grain sowing and experimental design

**Fig. 1:**
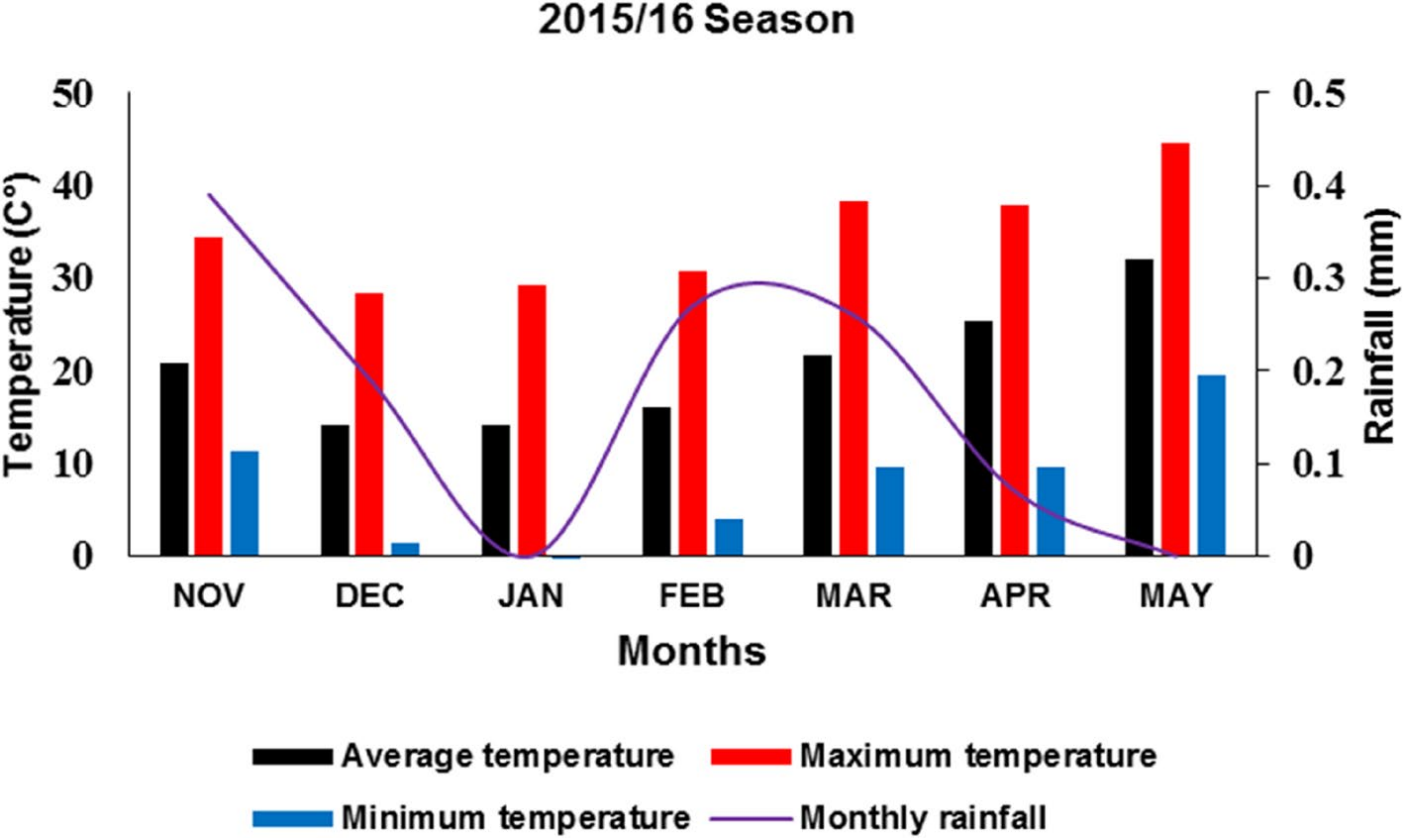
The average monthly rainfall in the location of Saudi Arabia (24°25’34.43” N, 46°39’10.86” E) during the growing season 2015/2016

The 12 genotypes' grains were sown in the early winter (1^st.^ November 2015/2016) at the site of the Experimental Research Station for one growing year, King Saud University, Dirab, 35 km southwest of Riyadh, Saudi Arabia (24°25’34.43” N, 46°39’10.86” E). This region has an arid climate, with normal highs and lows of 14.15 to 32 °C during the growing season (Table 2 and Fig. 1). In the period from 2015 to 2016, the monthly average rainfall varied between 0 and 0.39 mm (Table 2 and Fig. 1). Table 3 depicts the physical and chemical characteristics of the soil during the growing season. An experimental soil was created by extracting the top 20 cm layer of loamy sand from uncultivated land. Prior to planting, the ground was soaked twice with fresh water. A block of land was leveled, and a uniform soil type was selected to reduce environmental variation. Grains of each genotype were hand-planted in two rows per plot (30 cm apart and 2 meters long). The rows’ exact grain spacing was thirty centimeters. Six replicates of a randomized complete block design (RCBD) were used. Twelve genotypes were randomly distributed within each block. Standard agricultural practices were consistently applied.

**Table 2:** Weather information for the experimental site, including the means of maximum, minimum, and average temperatures, as well as the monthly total rainfall during the 2015–2016 seasons

PARAMETER	Average temperature	Maximum temperature	Minimum temperature	Monthly rainfall
NOV	20.82	34.48	11.37	0.39
DEC	14.15	28.36	1.38	0.19
JAN	14.17	29.28	-0.45	0.00
FEB	16.14	30.73	3.95	0.27
MAR	21.76	38.26	9.55	0.26
APR	25.31	37.94	9.54	0.07
MAY	32.00	44.52	19.48	0.00

**Table 3:** Basic descriptions of the experimental soil

**Parameters**	**Value**
Clay percentage, %	9.0
Silt percentage, %	10.0
Sand percentage, %	81.0
pH	8.0
Total N percentage, %	0.9
CaCO3 percentage, %	9.1

## Data recording and statistical analysis

The heading date (DH) at 50% anthesis and the aboveground main shoot's plant height (PH) at maturity were recorded. Physiological parameters were measured during the growing season and after maturity. The filling phase (FP) was determined in days, considering that around 50% of the plants produced grains during this period. The leaf area (LA in cm^2^) was recorded at the filling stage. In order to calculate the agronomic yield, the plants were manually harvested from 0.6 m^2^, or the whole plot area, during the two growing seasons after reaching maturity. The date of maturity (DM) was recorded, and five spikes were separated to measure weight (SPW in grams), spike number (SPN), and spike length (SPL in centimeters). The grain number (GN) was determined using a Seedburo 801 Count-A-Pak (Seedburo). The spike number/m^2^ (SN) and weight/m^2^ (SW) were also determined. To calculate the 100-grain weight (GW in grams), a total of 100 grains were weighed and counted. The grain yield (GY; kg/ha) was calculated after the grain was threshed from the biomass.

In contrast, the plot area's total aboveground biomass (straw and grain) was manually harvested and sun-dried and subsequently calculated as the biological yield (BY in kg/hectare) weight. The following formula was used to estimate the percentage of (HI) HI% = (GY∕BY) × 100. The data from the growing seasons were statistically analyzed using the ANOVA test for complete randomized block design (RCBD) using the SAS program (1985). The means of the barley genotypes were compared using the least significant difference values at two probability levels (0.05 and 0.01).

## DNA sampling and ISTR, SRAP, TRAP, and SSR markers amplification

### Molecular analysis

Molecular experiments were achieved at the Genetics and Cytology Department, Biotechnology Research Institute, National Research Center (NRC), Dokki, Giza, Egypt. The DNA extraction from the barley plants was performed using a Wizard Genomic DNA Purification Kit (Promega Corporation Biotechnology, Madison, WI, USA). The isolated DNA was then treated with RNase and kept at -20°C. Prior to conducting the ISTR, SRAP, TRAP, and SSR assays, the DNA was diluted to a concentration of 25 ng/μl. Twenty-three ISTR primers [21], 22 SRAP primers [22], 25 TRAP primers [22], and 30 SSR markers [23] were used in the experiment (supplementary data Tables S1, S2, S3, and S4) to study genetic variation of the barley genotypes. The PCR mixture (10.00 μL) contained 50 ng of DNA from the genome, 1× PCR buffer, 1.5 mM MgCl2, 0.1 mM each dNTP, 0.5 M each of the forward and reverse primers, and 1 U of Taq polymerase. PCR was performed using a T1 thermocycler (Biotech Company, Germany). The ISTR analysis was performed under specific settings for the PCR cycle, which included a 5-minute incubation at 94°C, followed by five cycles consisting of 1 minute at 94°C, 1 minute at 35°C, and 1 minute and 40 seconds at 72°C. This was then followed by 35 cycles with the same parameters, except for the annealing temperature, which was set at 50°C. Finally, there was a 7-minute incubation at 72°C to complete the PCR cycle [24]. For the SRAP and TRAP programs, the first denaturation at 94°C for 3 minutes was followed by 35 cycles of denaturation at 94°C for 1 minute, annealing at 50°C and 55°C (depending on SSR primers) for 1 minute. Subsequently, the extension was performed at 72°C for 2 minutes, followed by a final extension at 72°C for 10 minutes. The amplified PCR products were separated on a 2-3% (w/v) agarose gel in TBE buffer containing 0.1 g/cm3 ethidium bromide. Following electrophoresis, a picture of the gel was taken with a UV transilluminator. After removing unreproducible bands, the ISTR, SRAP, TRAP, and SSR data were scored based on each primer's presence (1) or absence (0).

## Statistical analysis

### Data from molecular markers and genetic variation

According to Nei and Li [25], a similarity matrix was calculated using molecular marker data as follows:

$$SM = {2N}_{ij}/({N}_{i}+{N}_{j})$$

Where *Nij* represents the number of alleles found in both the *ith* and *jth* genotypes, *Ni* represents the number of bands found in the *ith* genotype, and *Nj* represents the number of alleles found in the *jth* genotype. Subsequently, the rate unweighted pair group method with arithmetic average (UPGMA) grouping technique was used for the similarity matrix. The coordinates were obtained using the principle coordinate analysis (PCoA) similarity matrix, which serves as an alternative to hierarchical clustering. These locations were then utilized to generate scatter plots depicting the genotype relationships. PAST version 1.62 was used for both UPGMA and PCoA [26]. Furthermore, 1000 simulations were run using PAUP* version 4.0.b5 to validate the dendrogram’s reliability [27]. The potential correlation between molecular and morphological data was evaluated by a Mantel test using PAST software, version 4.11 [28].

### Marker efficiency analysis

The EMC program, a crucial tool in primer performance evaluation, was utilized in this study. It computed various metrics for each primer, such as the polymorphic information content (PIC), discriminating power (DP), and predicted heterozygosity (H). The PIC was calculated using the formula: *PIC = 1– Σ pi*^*2*^* – Σ Σ pi*^*2,*^ where *pi* and *pj* are the population frequencies of the *ith* and *jth* alleles, respectively. The first summation represents the entire number of alleles, whereas the two following summations represent all the i and j where *i = j* [29, 30].

E (EMR) was determined using the formula [31] *EMR = n β*, where *n* is the average number of fragments amplified by an individual to a specific system marker (multiplex ratio). In addition, *β* is estimated from the number of polymorphic loci (*np*) and the number of nonpolymorphic loci (*nnp*); *β* = *n*_*p*_/ (*n*_*p*_*+ nnp*).

The marker index, a crucial component in our primer evaluation, was calculated using the formula: *MI = E H*_*avp*_ [31]. It is the product of the effective multiplex ratio and the average expected heterozygosity for polymorphic markers, where *H* denotes the average expected heterozygosity for the polymorphic markers. It is also equal to *Σ H*_*p*_*/n*_p_, where the summation is over all polymorphic sites with *H*_*p*_ and *n*_*p*_ defined as above.

Discriminating power was calculated using the formula [32] *DP = 1 – C*, the probability that two randomly chosen individuals exhibit different banding patterns and are thus distinguishable. *C* is defined as the confusion probability. For the *ith* pattern of the given *jth* primer, present at frequency *p*_*i*_ in a set of varieties, the confusion probability is *C = Σ c*_*i*_*= Σ p*_*i*_(*N*_*pi*_*−1*)/(*N−1*), where for *N* individuals, *C* is equal to the sum of all *c* for all of the patterns generated by the primer.

The calculation of expected heterozygosity was performed using Liu's formula [33] *H = 1 – Σ p2*, the probability that an individual is heterozygous for the locus in the population. *P* is the allele frequency for the *ith* allele, and the summation is over all present alleles.

### STRUCTURE analysis

An analysis was conducted using data from 100 different molecular markers, including 25 TRAP, 22 SRAP, 23 ISTR, and 30 SSR markers. The purpose of this analysis was to determine the number of subgroups that may explain the population structure. The admixture model-based clustering approach was used in the software program STRUCTURE 2.3.3 to analyze population structure. The burn-in parameters of 15,000 and 15,000 MCMC replications were used for structural analysis. The Structure Harvester program was used to calculate the proper number (K) of subgroups. K was evaluated on a one-to-ten scale, with three iterations for each group.

## Results

### Agro-morphological characterization of barley genotypes

Analysis of variance revealed that the means of the morphological traits harvest index (HI) and grain yield (GY) significantly differed among the barley genotypes. In addition, highly significant differences were obtained among the studied barley genotypes for the remaining morphological characteristics under study (Table 4). No significant differences were found among the three replicates for any of the morphological features, as determined by the variance analysis.

**Table 4:** Analysis of variance of 15 morphological traits of the barley genotypes under study

S.O.V.	DF	FP	DM	DH	LA	SPN	SPW	GN	GW
Model	13	437**	394**	422**	1301**	615**	4.23**	930**	1092**
Genotypes	2	436**	393**	420**	1297**	614**	4.20**	920**	1091.5**
Reps	11	1n.s	1n.s	2n.s	4n.s	1n.s	0.03n.s	10n.s	0.5n.s
Error	22	13	51	57	207	154	0.74	239	162
Total	35	450	445	479	1508	769	4.97	1169	1254
**Continue**									
**S.O.V.**	**DF**	**SPL**	**PH**	**SN**	**HI**	**SW**	**BY**	**GY**	
Model	13	42**	3569**	246011**	0.059*	1331893**	304**	29.29*	
Genotypes	2	41**	3558**	245722**	0.059*	1313138**	300.85**	29.25*	
Reps	11	1n.s	11n.s	289n.s	0.000	18755n.s	3.15n.s	0.03n.s	
Error	22	11	262	61844	0.045	480399	67	22.18	
Total	35	53	3831	307855	0.105	1812293	371	51.46	

** and * indicate statistical significance at the 0.01 and 0.05 probability levels, respectively; *n.s.* Not significant, *DF* Degree of freedom, *FP* Filling Period, *DM* Date of Maturity, *DH* Date of Heading, *LA* Leaf Area, *SPN* Spike Number/plant, *SPW* Spike Weight/plant, *GN* Grain Number/spike, *GW* 100-Grain Weight in grams, *SPL* Spike Length, *PH* Plant Height; *SN* Spike Number/m2, *HI* Harvest Index (Grain yield/ Biological yield), *SW* Spike Weight/ m2, *BY* Biological yield (Ton/Hectar), *GY* Grain Yield (Ton/Hectar)

Comparisons between the mean values of morphological traits for the different genotypes are presented in Table 5. Based on this comparison, the KSU-B101 genotype surpassed the other genotypes in five morphological traits (i.e., spike number/plant (40.3), 100-grain weight (56.17), spike number/m^2^ (806.67), harvest index (0.368) and grain yield (7.81)). Moreover, the Sahrawy genotype had the highest significant values overall for all genotypes in another five morphological traits (leaf area (36.2), spike weight/plant (3.3), spike length (9.8), spike weight/m^2^ (1676.3) and biological yield (30.7). Conversely, the Gusto genotype exhibited the lowest values for three morphological traits: spike length (6.5), plant height (67.6), and grain weight per plant (38.8). The Sahrawy genotype exhibited the lowest values for three traits: days to grain filling (34), plant height (70.3), and grain number/m2 (46). Additionally, the KSU-B105 genotype displayed low values for three traits: leaf area (18.47), harvest index (0.218), and grain yield (5.18). The KSU B105 cultivar demonstrated superior performance compared to other cultivars in terms of heading, maturity date, and grain number per spike (Table 5).

**Table 5:** Comparisons of morphological trait means (using Tukey’s LSD values) among the barley genotypes under study

Genotype	FP	DM	DH	LA	SPN	SPW	GN	GW
KSU B101	38.3def	112bc	73.67cde	23.4bcde	40.3a	2.8bcde	58abc	56.17a
KSU B102	46.3a	112bc	65.67g	20.4cde	30cde	2.5ef	49def	54.7a
Giza 124	41.3c	111.67bc	70.3f	25.4bc	32.3bcd	2.7cdef	58.67ab	41.67de
Gustoe	39d	111.3bc	72.3def	18.57e	27.3ef	2.4f	53cde	38.8e
KSU B103	36.67g	111.67bc	75bcd	20.17de	25f	3bc	54.67bc	49.9b
Giza 121	37.3fg	108.3d	71ef	19.5de	34.3bc	3.07ab	56.3bc	47.37bc
KSU B104	38.67de	109.67cd	71ef	24.57bcd	33.3bc	2.9bcd	47.67ef	48.1bc
Giza 126	37.67efg	112.67b	75bcd	26.6b	34.67b	1.97g	46.3f	48.07bc
Sahrawy	34h	111bc	77ab	36.2a	27ef	3.3a	46f	41.77de
KSU B105	41c	119.3a	78.3a	18.47e	33.3bc	2.4f	62.67a	45.1cd
Asser	44b	119.67a	75.67abc	37a	28def	2.67def	55.3bc	47.7bc
Giza 123	34.3h	111bc	76.67ab	24.27bcd	28.67def	2.6def	54.3bcd	56.7a
LSD	1.28	2.58	2.73	5.2	4.49	0.31	5.58	4.59
**Continue**								
**Genotype**	**SPL**	**PH**	**SN**	**HI**	**SW**	**BY**	**GY**	
KSU B101	8cd	73f	806.67a	0.368a	936.7e	21.19de	7.81a	
KSU B102	9.67ab	91bcd	600cde	0.264bcd	1252.3bcd	24.29c	6.39abcd	
Giza 124	6.8de	91.67bc	646.67bcd	0.285bcd	1284bcd	25.7bc	7.37ab	
Gustoe	6.5e	67.6f	546.67ef	0.326ab	1175.2cde	24.76c	7.97a	
KSU B103	8.5bc	83e	500f	0.296abc	1242.7bcd	25.24c	7.48ab	
Giza 121	8cd	96.3ab	686.67bc	0.266bcd	1477ab	28.57ab	7.47ab	
KSU B104	6.8de	85.67de	666.67bc	0.28629bcd	1047de	20.95de	5.99bcd	
Giza 126	9.5ab	98a	693.3b	0.281bcd	1247bcd	24.76c	6.95abc	
Sahrawy	9.8a	70.3f	540ef	0.222cd	1676.3a	30.7a	6.77abcd	
KSU B105	7.5cde	89cd	666.67bc	0.218d	1304.3bc	23.8cd	5.18d	
Asser	8.17c	73.3f	560def	0.237cd	1361bc	25.48c	6.03bcd	
Giza 123	8cd	87.67cde	573.3def	0.265bcd	1042.3de	20.24e	5.35cd	
LSD	1.19	5.84	89.78	0.07	250.22	2.95	1.7	

*FP* Filling Period, *DM* Date of Maturity, *DH* Date of Heading, *LA* Leaf Area, *SPN* Spike Number/plant, *SPW* Spike Weight/plant, *GN* Grain Number/spike, *GW* 100-Grain Weight in grams, *SPL* Spike Length, *PH* Plant Height, *SN* Spike Number/m2, *HI* Harvest Index (Grain yield/ Biological yield), *SW* Spike Weight/ m2, *BY* Biological yield (Ton/Hectar), *GY* Grain Yield (Ton/Hectar). Means connected with the same letter (in each trait) are not significantly different

## Molecular characterization of barley genotypes via ISTR, SRAP, TRAP, and SSR analysis

### ISTR analysis

In this study, 23 ISTR primer pairs were used. A total of 115 bands were obtained from these markers (Fig S1, Tables S3, and Table 6). The P3-F3B3 marker produced the highest number of bands (9 bands). The markers P18-F8B10 and P22-F9B7 exhibited a total of 8 bands apiece, while the markers P2-F2B10 and P19-F9B3 had the lowest count of 2 bands. The average number of bands per marker was 5.00. The ISTR markers display 82 polymorphic bands, with an average of 3.65 bands per marker, accounting for 94.3% of all bands (Table S5 and Table 6 for additional information).

**Table 6:** Marker efficiency analysis (MEA) of 100 markers (23 ISTR, 22 SRAP, 25 TRAP, and 30 SSR) applied on twelve barley genotypes

Source of primers	No. of primers	BS	PB	A/L		SC		PIC value		DP	
				Range	Main	Range	Main	Range	Main	Range	Main
ISTR	22	115	82	2 to 9	3.6	0.53 to 0.87	0.70	0.24 to 0.47	0.403	0.67 to 0.85	0.40
SRAP	22	232	202	3 to 18	9.2	0.29 to 0.74	0.56	0.47 to 0.13	0.44	0.47 to 0.5	0.397
TRAP	25	180	100	2 to 14	4.0	0.60 to 0.88	0.74	0.13 to 0.47	0.379	0.09 to 0.51	0.346
SSR	30	62	51	1to 4	2.066	0.40 to 0.85	0.58	0.25 to 0.472	0.435	0.0 to 0.923	0.627
Combination	99	51	62	1 to 4	1.215	0.32 to 0.89	0.687	--	--	--	--

*SB* Scored bands, *PB* Polymorphic bands, *A/L* Allele/ locus, *SC* Similarity coefficient, *PIC* Polymorphic information content, *DP* Discriminating power

### SRAP analysis

The calculation of the polymorphic information content (PIC) for 22 SRAP primers resulted in the determination of their discriminating power (DP). A total of 232 bands were amplified from the 12 barley genotypes using 22 SRAP markers. The mean number of amplified bands per marker was 10.54, ranging from 3 bands, such as the “Me5Em10” marker, to 18 bands, like the “Me1Em7” marker. SRAP markers produced 232 bands, with 202 polymorphic bands with an average of 9.2 per marker and accounting for 87.07% of the total bands. The amplified bands exhibited a size range of 100 to 1000 bp (Figure S1, Table S4, and Table 6).

### TRAP analysis

Twenty-five primer pairs of TRAP markers were utilized (Fig S1, Table S7, and Table 6). The T11 marker exhibited the highest number of bands, with a total of 14 bands. It was followed by the T10 marking, which had 11 bands. In contrast, the T4 marker displayed the lowest number of bands, with only two bands. The mean number of bands per marker was 7.20. The TRAP markers generated a total of 180 bands, out of which 100 were polymorphic. The average number of polymorphic bands per marker was 4.0, representing 55.56% of all the bands (Tables S5 and 6).

### SSR analysis

Thirty sets of primer pairs of SSR markers were used, producing a total of 62 bands (Figure S1, Table S6, and Table 6). The SSR marker bmac0297 displayed the highest number of bands (4 bands). Subsequently, there were two markers, namely bmag0013 and bmac0127, each of which possessed three bands. Conversely, the SSR markers bmag0841, bmag0115, and bmac0282 exhibited the lowest number of bands, each having only one band. Each primer, on average, displayed 2.06 bands. The SSR markers generated 51 polymorphic bands, with an average of 1.7 bands per marker. The polymorphic bands represented 82.26% of all the bands (Tables S6 and 6).

### Marker efficiency analysis (MEA)

The EMC is a simple tool for estimating the efficacy of specific marker polymorphisms. Tables S3, S4, S5, S6, and 6 depict the polymorphism indices of the selected ISTR, SRAP, TRAP, and SSR markers. The PIC serves as an indicator of the diversification and incidence of alleles generated among barley genotypes for each marker.

The average heterozygosity (H) for each ISTR marker ranged from 0.272 (P4-F3B5 and P14-F8B5) to 0.568 (P13-F8B3, P21-F9B6, and P22-F9B7). The mean PIC for the analysis of the ISTR markers in MEA was 0.403. The highest value was observed with three markers (P13-F8B3, P21-F9B6, and P22-F9B7), reaching 0.472. The value of 0.471 was observed for two markers, namely P2-F2B10 and P20-F9B5. The effectiveness of the ISTR marker system on different barley genotypes was evaluated by determining the marker index (MI). The MI was determined to be the highest among three markers (P13-F8B3, P21-F9B6, and P22-F9B7), with a value of 0.568. The marker P20-F9B5 had a slightly lower MI value of 0.566. The markers P4-F3B5 and P14-F8B5 both had the lowest MI value of 0.272. To evaluate the efficacy of the ISTR marker, we determined its DP by averaging a DP value of 0.394, with a range from 0.067 (P8-F5B3) to 0.846 (P2-F2B10). The effective multiplex ratio (E) was consistently 1 for all ISTR markers, also referred to as "EMR." The variation is attributed to the polymorphic locus component of an individual screening. With the exception of ISTR (or other codominant markers), the value of E is one because each assay reveals a single locus. The correlation coefficients between PIC and MI (*r* = 0.998, *p* ≤ 0.05), DP and PIC (*r* = 0.87, *p* ≤ 0.05), PIC and H (*r* = 0.99, *p* ≤ 0.05), and MI and D (*r* = 0.75, *p* ≤ 0.05) were all statistically significant and positive.

The mean heterozygosity (H) per SRAP markers ranged from 0.142 (Me5Em10) to 0.568 (Me1Em5, Me5Em9, and Me6Em12). The average PIC for MEA analysis of the SRAP markers was 0.442, with the highest value being 0.472 for four markers (Me1Em5, Me5Em4, Me5Em9, and Me6Em2) and 0.470 for three markers (Me1Em7, Me1Em11, and Me6Em4). The marker index (MI) was calculated to estimate the effectiveness of the SRAP marker system on barley genotypes. Additionally, it was found to be highest for three markers (Me1Em5, Me5Em9, and Me6Em12) (MI = 0.568), followed by Me5Em4 and Me6Em2 (MI = 0.567), and lowest for the marker (Me5Em10) (MI = 0.142). We estimated DP using a mean index of DP = 0. 397, which ranging from 0.00 (Me5Em10) and 0.504 to determine the prudent profile of the SRAP marker (Me6Em4). The effective multiplex ratio (E) was defined, also known as “EMR,” and had a value of 1. This difference is due to the individual screening of polymorphic loci. With the exception of SRAP (or other codominant markers), the value of E is 1 because each assay exposes a single locus. Significant positive correlations were detected between PIC and MI (*r* = 0.990, *p* ≤ 0.05), DP and PIC (*r* = 0.94, p ≤ 0.05), PIC and H (*r* = 0.99, *p* ≤ 0.05), and between MI and DP (*r* = 0.938, *p* ≤ 0.05).

For the TRAP markers, the average heterozygosity (H) per marker ranged from 0.142 (T3 and T4) to 0.568 (T23). The average PIC for the TRAP markers was 0.379. The highest PIC values were observed for two markers, T22 and T23, with a value of 0.472. The marker index (MI) was used to estimate the efficacy of the TRAP markers system on barley genotypes, and the maximum MI was found for one marker (T23) (MI = 0.568), followed by T22 (MI = 0.567), and the minimum MI was found for two markers (T3 and T4) (MI = 0.142). To determine the prudent profile of the TRAP markers, we estimated discriminative power (DP) using a mean index of DP = 0. 346, ranging between 0.00 (T3 and T4) and 0.508 (T5). The effective multiplex ratio (E) had a numerical value of 1. The variation observed is attributed to the presence of the polymorphic locus in individual testing. Except for TRAP (or other codominant markers), the value of E is 1 because each assay uncovers a solitary locus. The correlation coefficients between PIC and MI (*r* = 0.997, *p* < 0.05), DP and PIC (*r* = 0.992, *p* < 0.05), PIC and H (*r* = 0.99, *p* < 0.05), and MI and DP (*r* = 0.992, *p* < 0.05) were all statistically significant and positively correlated.

### Cluster analysis

**Fig. 2:**
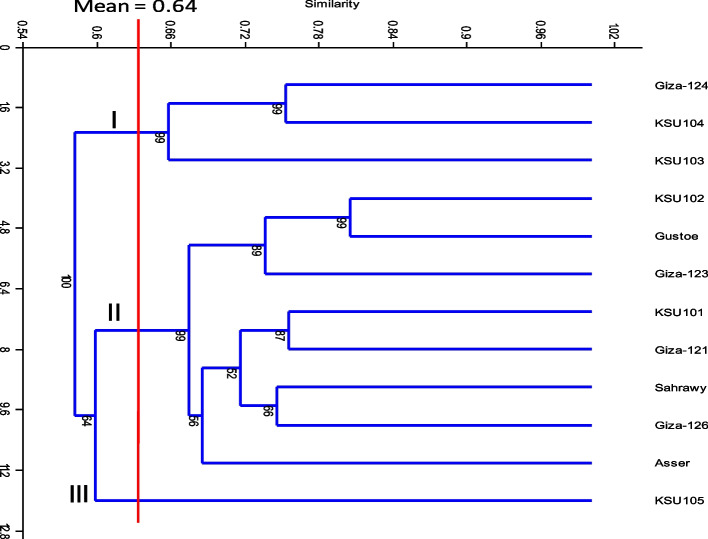
A dendrogram depicting the genetic relationships among 12 barley genotypes derived from allelic data of a combined analysis of 100 markers (23 ISTR, 22 SRAP, 25 TRAP, and 30 SSR) using a similarity coefficient

The ISTR, SRAP, TRAP, and SSR datasets were combined to assess the genetic correlation across different barley genotypes. The similarity coefficients calculated using the combined ISTR, SRAP, TRAP, and SSR data varied between 0.48 and 0.80, with an average of 0.64 for all twelve barley genotypes. The genetic distance between KSU102 and the Gustoe genotypes was the smallest, measuring 0.80. The genetic distance between KSU101 and KSU105 was the highest, measuring 0.48 (Table S7). All the aforementioned findings indicated that the genotypes exhibited slight genetic variations. The dendrogram, produced using UPGMA, depicts the genetic association between the genotypes. The dendrogram and its similarity matrix had a correlation coefficient of 0.903, indicating that it may be a suitable depiction of the genetic association. With a similarity coefficient of 0.80, the dendrogram revealed that all the genotypes could be divided into three groups (Table S7 and Fig. 2).

The first group consisted of three genotypes (Giza-124, KSU104, and KSU103) with a bootstrap value of 99%. They share a pedigree, such as KSU104 and KSU103, which have the same parent as C.C.89, and Giza 124 also enters the lineage as KSU104. Cluster II consisted of eight genotypes divided into three subgroups. The genotypes were identified in the first subgroup, which had an 89% bootstrap value. The KSU102, Gustoe, and Giza-123 genotypes were found to share the pedigree genotype KSU102. The second subgroup had a bootstrap value of 52% and included four genotypes (KSU101, Giza-121, Sahrawy, and Giza-126). The Giza-121 genotype was shown to share the pedigree genotype KSU101, which has a pedigree with Giza-121, and Sahrawy, which shares a parent with Baladi-16. Only one genotype was present in the third subgroup, which had a bootstrap value of 56% (Asser). The third group consisted of a single genotype (KSU105) with a bootstrap value of 64%. The pedigree of the Rihane X Lignee variant exhibited distinct differences compared to the other genotypes (Fig. 2).

**Fig. 3:**
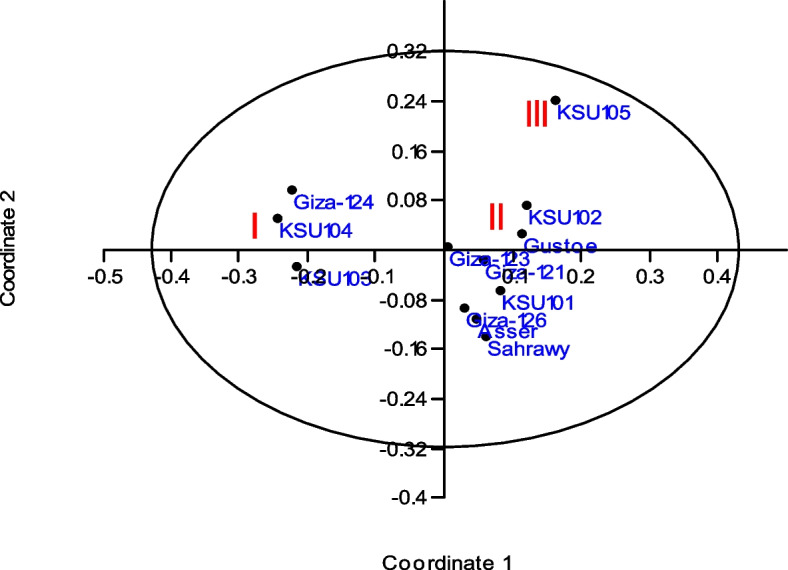
PCoA of 12 barley genotypes with 100 polymorphic ISTR, SRAP, TRAP, and SSR combinations

The PCoA results align with the UPGMA clustering analysis. The dendrogram grouping corresponded to the scatter plot grouping (Fig. 3). The PCoA also classified all the genotypes into three groups. The first two principal coordinates accounted for 46.99% of the total variation (30.43 and 16.56% of the first and second principal coordinates, respectively).

### Structure analysis

**Fig. 4:**
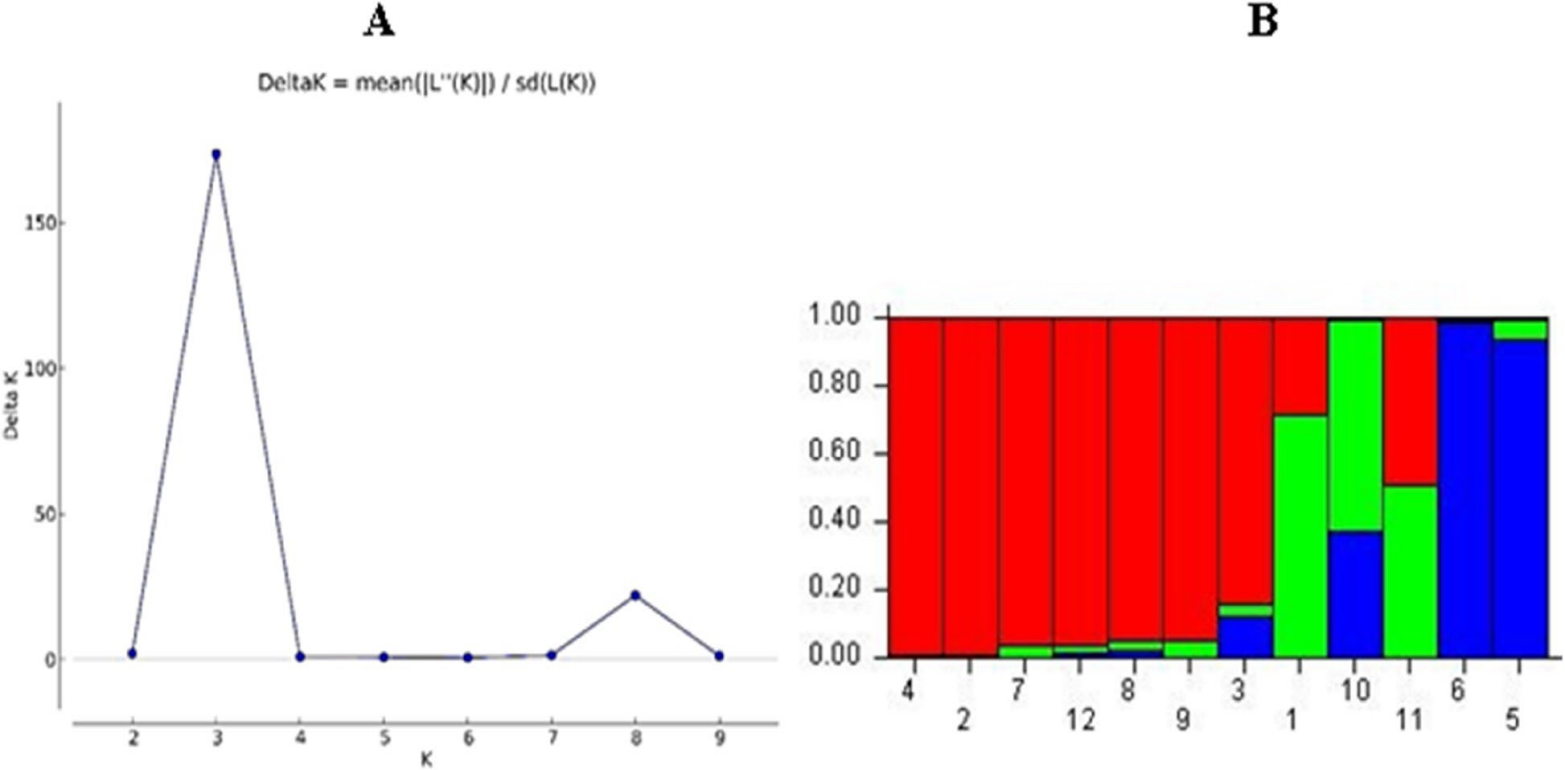
Genetic organization derived from the Bayesian grouping of twelve barley genotypes; Panel A, with ΔK values; Panel B, with genetic clustering computed (K = 3) utilizing Structure 2.3.3 software to display three primary populations

Structure 2.3.3 was used to infer the population structure of the twelve barley cultivars. The peak of delta K was observed at K = 3, indicating the presence of three main groups (Fig. 4A). The 12 barley genotypes were categorized into the main clusters and admixtures. Subgroup one had seven genotypes: 4, 2, 6, 3, 12, 9, and 5 (Gustoe, KSU102, KSU104, Giza-123, Giza-126, Sahrawy, and Giza-124). Subgroup two had three genotypes: 7, 1, and 11 (KSU101, KSU105, and Asser). Subgroup three had two genotypes: 8 and 10 (Giza-121 and KSU103, Fig. 4B).

## Discussion

Barley genotypes were shown to have highly significant variations in their agronomic traits when examined in arid conditions. These differences were evident in different aspects, such as yield components, grain yield, biological yield traits, and foliage (Table 4). Notably, the genotype KSU-B101 outperformed all others in five key yield-related and yield-component traits, suggesting its suitability as a high-yield potential cultivar for cultivation in marginal (arid) areas (Table 5). Conversely, the Sahrawy cultivar exhibited superiority in five distinct traits, particularly leaf area and biological yield, indicating its potential suitability for animal feed purposes. Interestingly, the KSU B105 cultivar demonstrated significant values for early maturity traits such as date of heading and maturity.

Additionally, it exhibited a high grain number per spike, implying its potential as a dual-purpose cultivar for animal feed and human food in arid regions. In contrast, Roohi et al. examined the agronomic performance of barley genotypes under rainy conditions in western Iran. The study revealed significant genotypic differences in terms of grain yield and yield components, except for the 1000-kernel weight [34].

Overall, our findings suggest that dominance plays a significant role in determining the observed traits. This finding is consistent with previous studies, such as Ghandorah et al., which emphasized dominance deviation over additive variance for various traits [19]. Similarly, El-Naggar et al. investigated the genetic variance components and gene action controlling yield and its components in barley under normal and water stress conditions. The study's findings suggested that dominance and associated nonallelic interactions significantly influenced these characteristics, surpassing the effects of additive and additive dominance within [35].

Molecular markers have demonstrated their efficacy as tools for studying genetic variation. The results of our research are consistent with previous studies, such as the one conducted by Powell et al.. They used cluster analysis to identify commonalities among 19 accessions and confirmed their genetic study by utilizing SRAPs, ISTRs, and ISSRs to examine systematic relationships between two families and determine molecular phylogeny [31]. Consistent with these findings, our study highlighted the superior differentiation capacity of ISTR markers in conjunction with SRAP markers for describing genetic variation, exhibiting numerous polymorphic markers per reaction [36]. Furthermore, SSRs demonstrated an average of 8.27 alleles per locus, indicating their robust polymorphic nature. In comparison, ISTR and AFLP markers exhibited relatively lower allele counts, with values of 1.39 and 1.42, respectively [37].

Nonetheless, all three molecular markers, ISTR, SSR, and AFLP, proved remarkably polymorphic and effective in distinguishing avocado accessions, with ISTR and AFLP techniques yielding particularly promising results [37]. Notably, ISTR markers exhibited the highest degree of polymorphism compared to AFLP and SSR markers in specific tested individuals [37]. In a study conducted by Torres-Morán et al., they identified 94 loci in Roselle genotypes using ISTR markers, with 80 (85%) being polymorphic, highlighting the effectivity of this marker type [38]. Additionally, a comparative assessment of 24 ISTR, 16 ISSR, and 30 SRAP primer combinations revealed the superiority of the SRAP assay in terms of resolution, effectiveness of selective capacity, and level of genetic variation. This assay provided a deeper understanding of the total number of viable alleles and polymorphic amplicons. Despite the moderate level of variation observed among the investigated types, the ISTR profile yielded valuable data for the study [39]. Overall, our study and related research emphasize the high levels of polymorphism exhibited by various marker types. Specifically, SRAP markers consistently generate more polymorphic bands than SCoT, CDDP, and CBDP markers, emphasizing their utility in genetic variation studies [40].

The Polymorphic Information Content (PIC) values exhibited consistency across all markers, averaging 0.48. However, differences in the average heterozygosity, which indicates genetic variation, were observed among different markers, with RAPD showing 0.43, ISSR 0.45, AFLP 0.47, and ISTR 0.36 [41]. In a comparative analysis of 24 ISTR, 16 ISSR, and 30 SRAP primer combinations, a PIC value of 0.94 was reported. Additionally, the study reported an assay efficiency index of 47.04, an effective multiples ratio of 10.04, and a marker index (MI) of 9.74 [39].

The MI is recognized as a suitable measure of marker efficacy [14], and it indicated a 1.18-fold higher MI for ISTR than for SRAP or ISSR. This emphasizes the distinctiveness of the ISTR assay, which is attributed to its higher EMR and assay efficiency index values [42]. Studies have consistently highlighted the discriminatory ability of retrotransposon markers like ISTRs, known for identifying numerous polymorphic loci per individual response [43].

Utilizing 24 SSR markers revealed reduced allelic heterozygosity but enhanced primer specificity, with an average PIC of 0.239 and an average MI of 0.005 [44]. Similarly, the efficacy of twelve ISSR primers was evaluated, revealing an average PIC of 0.361 and MI of 0.016, with varying levels of primer discriminating power [45]. Additionally, Scot markers demonstrated average PIC values of 0.33, while CDDP and CBDP markers exhibited values of 0.37 each, with SRAP markers showing a slightly lower average PIC of 0.31. Comparatively, the MI of SRAP and CBDP markers was higher than that of SCoT and CDDP markers [46].

These findings highlight the informative nature of ISSR and SSR marker systems. ISSRs showed an average anticipated heterozygosity (Hexp) of 0.264 and SSRs of 0.457 [47]. The variations in PIC values among different marker types highlight their distinctive capacity to study genetic variations, providing valuable insights into the genetic makeup of the studied populations.

Both cluster analysis and Principal Coordinate Analysis (PCoA) revealed significant variation among barley genotypes. Clustering using SSR and morphological data enabled easy differentiation of genotypes by type (local landraces vs. variety), row number, and end-use. Notably, grouping based on both morphological and SSR data was notably consistent among 26 barley samples, where 15 SSR markers were employed [48]. In another study, SSR markers were utilized to explore the diversity of 103 wild barley genotypes from various locations in Jordan and 29 farmed barley genotypes. The analysis revealed clustered populations based on ecological and geographical factors [49].

Ten different barley genotypes were characterized using a combination of seven SSR markers and three SCoT primers. This analysis generated distinct dendrograms, with the dendrogram based on Triple-SCoT data exhibiting similarities to the SSR dendrogram [50]. Furthermore, clustering methodologies combining SSR and SNP genotypic data revealed three subpopulations among 153 barley genotypes, corroborating genetic investigations [51]. Other studies, such as Brbaklic et al. conducted a study categorizing breeding material into several groups using microsatellite, pedigree, and phenotypic data. The categorization was based on population structure, developmental features, and row type [52]. Moreover, systematic relationships between barley families were examined using ISSR, SRAP, and ISTR data to determine molecular phylogeny, with ISTR and SRAP markers showing good discriminating power for defining genetic variation [36]. Similarly, TRAP markers distinguished agricultural types effectively, while the SRAP marker dendrogram classified Egyptian barley cultivars into distinct groups based on genetic similarity coefficients [53–57].

The efficacy of clustering analysis was enhanced by combining data from several markers, such as SRAP, InDel, and ISSR. This revealed geographical and locational clustering among barley accessions [56]. Additionally, CDDP, CBDP, and SRAP markers, as well as SCoT markers, facilitated the clustering of barley genotypes into distinct groups, highlighting their utility in genetic research [40].

Clustering analyses using several molecular markers consistently yielded helpful data on the genetic variation and population structure of barley genotypes. This information is crucial for breeding and improvement programs. These findings underscore the importance of integrating molecular marker data with traditional morphological assessments to achieve comprehensive genotype characterization.

Based on the findings of the analysis, the population was divided into three distinct genetic groupings. At K = 3, these clusters (G1, G2, and G3) represented proportions of 34.9%, 86.3%, and 28.1%, respectively, under the non-admixture model. Notably, based on molecular data, Bayesian clustering analysis conducted using STRUCTURE software confirmed the groupings observed in both the UPGMA dendrogram and PCoA [55]. Additional investigation using K = 5 revealed the highest estimated likelihood [ln P (D)], suggesting the population could be partitioned into five clusters. The clusters consisted of distinct cultivars that were found to be unevenly distributed. Cluster 1 was mainly composed of cultivars like Rihane and Lemsi, while Cluster 2 featured cultivars like Kounouz and Manel [53]. Similarly, analysis by Mohammadi et al. indicated a peak delta K value at K = 3, supporting categorizing populations into three major subpopulations corresponding to Iranian landraces, foreign landraces, and varieties and advanced breeding lines [58].

In another study involving 48 barley accessions, clustering according to the admixture model revealed two distinct clusters. Cluster 1 consisted only of hulled barley accessions, whereas Cluster 2 consisted solely of hullless barley accessions. Cluster 2 could be further divided into three subclusters [56]. Furthermore, the examination of breeding material led to its categorization into three separate groups based on population structure. The genotypes were then classified based on their developmental habits and row type using principal coordinate analysis [52].

Similarly, an investigation utilizing 983 SNP markers identified the most likely number of subpopulations at K = 3, with Cluster 1 comprising 30.8% of accessions, Cluster 2 comprising 27.3%, and Cluster 3 comprising 41.9% [59]. Furthermore, the examination of the relative kinship among genotypes indicated minimal family structure, providing additional support against false-positive associations [60]. In contrast, a study involving 12 rice genotypes revealed a poor to non-existent population structure, with only two homogeneous groups identified at K = 2 [47]. These findings collectively demonstrate the utility of genetic clustering techniques in delineating population structure and understanding genetic variation within barley and other crop species.

## Conclusions

The KSU-B101 genotype outperformed the other genotypes in five morphological traits, whereas the Sahrawy genotype had the highest significant values overall of all genotypes in another five morphological traits. However, the cultivar KSU-B105 had the most significant differences in terms of date of heading, maturity date, and grain number per spike. Using combined ISTR, SRAP, TRAP, and SSR data, the similarity coefficients ranged from 0.48 to 0.80, with an average of 0.64 for all twelve barley genotypes. The dendrogram and its similarity matrix had a correlation coefficient of 0.903, indicating that the dendrogram may adequately depict the genetic association. The dendrogram indicated that all the genotypes could be classified into three groups, with a similarity value of 0.80. B101 has the potential to be cultivated as a high-yield cultivar in arid regions, whereas the Sahrawy cultivar is recommended for animal feed. However, KSU-B105 could be suggested as a purpose cultivar (i.e., for both animal feed and human food) in arid regions due to its early maturity and ability to produce a substantial number of grains per spike.

### Supplementary Information

Supplementary Material 1. 

## Data Availability

Not applicable.
