# Supplementary material for: Molecular and agro-morphological characterization of new barley genotypes in arid environments
Source: BMC Biotechnol. 2024 Jun 11;24:41. doi: 10.1186/s12896-024-00861-6 (PMC11167802; doi:10.1186/s12896-024-00861-6)
Supplement: Supplementary file 1 — Supplementary Material 1. [file 12896_2024_861_MOESM1_ESM.doc]

| **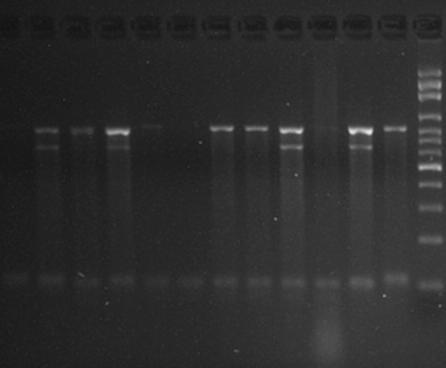** | **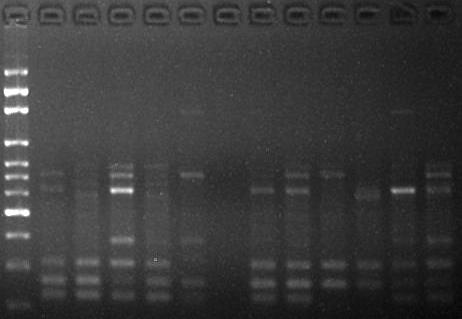** |
| --- | --- |
| **A** | **B** |
| **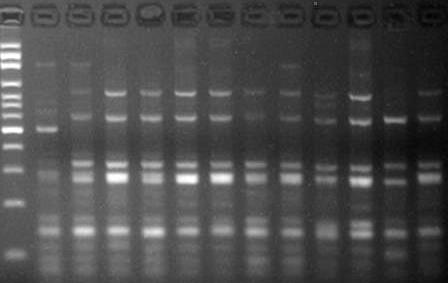** | **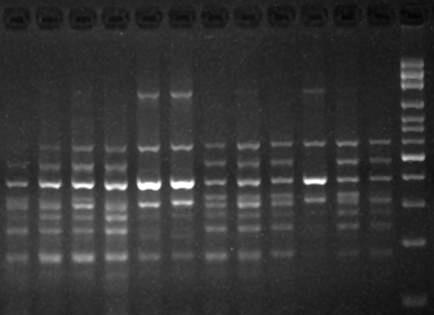** |
| **C** | **D** |

**Fig S1.** Amplification products using molecular markers techniques: (i.e.) SSR-bmag0105 (A), ISTR-F8B10 (B), SRAP-F3R4 (C), and TRAP-F10R3 (D).

**Table S1** Plant height (PH), number of effective tillers/plant (T/P), spike length (SL), and grain yield/plant (GY/P) average performance of parents and F2 in barley cultivated in 1992 season, [19]

| Genotype | PH (cm) | Spike Length (cm) | No. of active tillers/p | Grain Yield |
| --- | --- | --- | --- | --- |
| C. C 89 | 78.12 | 14.14 | 7.01 | 18.42 |
| Giza121 | 85.22 | 12.48 | 8.33 | 19.29 |
| Giza123 | 80.49 | 13.86 | 9.01 | 19.12 |
| Giza124 | 71.95 | 13.88 | 8.87 | 13.52 |
| Gustoe | 62.73 | 12.39 | 5.66 | 16.78 |
| C.C89 X Giza121 | 83.54 | 11.40 | 7.86 | 15.85 |
| C.C89 X Giza123 | 80.49 | 14.79 | 7.93 | 19.68 |
| C.C89 X Giza124 | 78.77 | 15.33 | 8.22 | 18.40 |
| C.C89 X Gustoe | 68.79 | 14.12 | 8.10 | 15.62 |
| Giza121 X Giza123 | 86.45 | 14.20 | 9.08 | 20.17 |
| Giza121 X Giza124 | 85.95 | 12.43 | 8.10 | 18.72 |
| Giza121 X Gustoe | 75.91 | 12.65 | 8.30 | 16.99 |
| Giza123 X Giza124 | 81.35 | 14.60 | 8.77 | 17.74 |
| Giza123 X Gustoe | 66.46 | 14.88 | 6.32 | 18.84 |
| Giza124 X Gustoe | 75.04 | 9.71 | 8.12 | 18.22 |
| Mean | 77.39 | 13.39 | 8.16 | 18.15 |
| L. S. D. (0.05) | 3.29 | 1.74 | 0.67 | 1.96 |

**Table S2** Estimates of genetic variance components in 5 x 5 barley diallel crosses, [19]

| Parameters | PH | No. of effective T/p | SL (cm) | GY/P (gm) |
| --- | --- | --- | --- | --- |
| D | 74.75 ±05.7 | 0.317 ±0.17 | 1.79 ±0.09 | 0.46 ±01.36 |
| F | - 27.38 ±28.89 | - 2.38 ±05.91 | 3.50 ±0.47 | - 1.87 ±0.629 |
| H1 | 177.67 ±66.29 | 27.38 ±14.04 | 5.11 ±1.09 | 29.78 ±15.87 |
| H2 | 129.95 ±61,19 | 24.71 ±12.53 | 3.23 ±1.00 | 28.42 ±14.65 |
| h2 | 705.83 ±41.5 | - 4.55 ±08.50 | 9.87 ±0.68 | - 5.82 ±09.94 |
| E | 1.36 ±02.55 | 0.38 ±00.52 | 0.06 ±0.04 | 0.48 ±00.52 |

D= Additive effects of genes, F= Covariance of dominance and additive effects, H1= Dominance effects of genes, H2= Dominance indicated asymmetry of positive, h2= The overall dominance effects of heterozygous loci, E= Error, *,** indicate significant and highly significant at 0.05 and 0.01 levels of probability, respectively.

**Table S3** Efficacy of 23 ISTR primers polymorphism calculated with iMEC of barley genotypes.

| ISTR | Sequences from 5' to 3' | | Tm °C | SB | PB | H | PIC | E | MI | D |
| --- | --- | --- | --- | --- | --- | --- | --- | --- | --- | --- |
| P1-F2B6 | F:AAAATGTAGTCTCTC | R:ATATATGGACTTAAGCAAGCA | 45 | 4 | 2 | 0.402 | 0.361 | 1.000 | 0.402 | 0.244 |
| P2-F2B10 | F:AAAATGTAGTCTCTC | R:GACCCTTTTGAAAACACATG | 45 | 2 | 2 | 0.565 | 0.471 | 1.000 | 0.565 | 0.846 |
| P3-F3B3 | F:GTCGACATGCCATCTTTC | R:ATTCCCATCTGCACCAAT | 45 | 9 | 5 | 0.445 | 0.393 | 1.000 | 0.445 | 0.346 |
| P4-F3B5 | F:GTCGACATGCCATCTTTC | R: CTTCTGTGAAAGTCCTAG | 45 | 3 | 1 | 0.272 | 0.255 | 1.000 | 0.272 | 0.154 |
| P5-F3B6 | F:GTCGACATGCCATCTTTC | R:ATATATGGACTTAAGCAAGCA | 45 | 4 | 2 | 0.328 | 0.303 | 1.000 | 0.328 | 0.179 |
| P6-F3B8 | F:GTCGACATGCCATCTTTC | R: CCTCCTTATTGGGAATGATAT | 45 | 7 | 7 | 0.527 | 0.448 | 1.000 | 0.527 | 0.374 |
| P7-F3B10 | F:GTCGACATGCCATCTTTC | R: GACCCTTTTGAAAACACATG | 45 | 7 | 5 | 0.451 | 0.397 | 1.000 | 0.451 | 0.366 |
| P8-F5B3 | F: ATATATGGACTTAAGCAAGC | R:ATTCCCATCTGCACCAAT | 45 | 6 | 4 | 0.252 | 0.237 | 1.000 | 0.252 | 0.067 |
| P9-F5B5 | F: ATATATGGACTTAAGCAAGC | R: CTTCTGTGAAAGTCCTAG | 45 | 4 | 2 | 0.479 | 0.416 | 1.000 | 0.479 | 0.449 |
| P10-F5B6 | F: ATATATGGACTTAAGCAAGC | R:ATATATGGACTTAAGCAAGCA | 45 | 3 | 2 | 0.535 | 0.452 | 1.000 | 0.535 | 0.462 |
| P11-F5B7 | F: ATATATGGACTTAAGCAAGC | R: GGAATATCATTCCCAATAAG | 45 | 3 | 2 | 0.462 | 0.404 | 1.000 | 0.462 | 0.462 |
| P12-F5B8 | F: ATATATGGACTTAAGCAAGC | R: CCTCCTTATTGGGAATGATAT | 45 | 6 | 4 | 0.449 | 0.396 | 1.000 | 0.449 | 0.344 |
| P13-F8B3 | F: TTGGACAACCATATTTTGACT | R:ATTCCCATCTGCACCAAT | 45 | 6 | 6 | 0.568 | 0.472 | 1.000 | 0.568 | 0.518 |
| P14-F8B5 | F: TTGGACAACCATATTTTGACT | R: CTTCTGTGAAAGTCCTAG | 45 | 3 | 2 | 0.272 | 0.255 | 1.000 | 0.272 | 0.103 |
| P15-F8B6 | F: TTGGACAACCATATTTTGACT | R:ATATATGGACTTAAGCAAGCA | 45 | 6 | 5 | 0.535 | 0.452 | 1.000 | 0.535 | 0.410 |
| P16-F8B7 | F: TTGGACAACCATATTTTGACT | R: GGAATATCATTCCCAATAAG | 45 | 8 | 5 | 0.556 | 0.465 | 1.000 | 0.556 | 0.495 |
| P17-F8B8 | F: TTGGACAACCATATTTTGACT | R: CCTCCTTATTGGGAATGATAT | 45 | 5 | 4 | 0.545 | 0.458 | 1.000 | 0.545 | 0.477 |
| P18-F8B10 | F: TTGGACAACCATATTTTGACT | R: GACCCTTTTGAAAACACATG | 45 | 8 | 7 | 0.501 | 0.431 | 1.000 | 0.501 | 0.376 |
| P19-F9B3 | F: ATATGGACTTAAGCAAGCCA | R:ATTCCCATCTGCACCAAT | 45 | 2 | 2 | 0.379 | 0.343 | 1.000 | 0.379 | 0.308 |
| P20-F9B5 | F: ATATGGACTTAAGCAAGCCA | R: CTTCTGTGAAAGTCCTAG | 45 | 5 | 3 | 0.566 | 0.471 | 1.000 | 0.566 | 0.523 |
| P21-F9B6 | F: ATATGGACTTAAGCAAGCCA | R:ATATATGGACTTAAGCAAGCA | 45 | 3 | 3 | 0.568 | 0.472 | 1.000 | 0.568 | 0.615 |
| P22-F9B7 | F: ATATGGACTTAAGCAAGCCA | R: GGAATATCATTCCCAATAAG | 45 | 8 | 7 | 0.568 | 0.472 | 1.000 | 0.568 | 0.484 |
| P23-F9B8 | F: ATATGGACTTAAGCAAGCCA | R: CCTCCTTATTGGGAATGATAT | 45 | 3 | 2 | 0.521 | 0.443 | 1.000 | 0.521 | 0.462 |
| Total |  |  |  | 115 | 82 | -- | -- | -- | -- | -- |
| Average |  |  |  | 5.00 | 3.565 | 0.467 | 0.403 | 1.00 | 0.467 | 0.394 |

F (forward), R (reverse), Tm (annealing temperature), SB (scored bands), PB (polymorphic bands), H (expected heterozygosity), PIC (polymorphism information content), E or EMR (Effective multiplex ratio), MI (marker index) and DP (discriminating power)

**Table S4** Efficacy of 22 SRAP primers polymorphism calculated with iMEC of barley genotypes.

|  | Primers | Sequences from 5' to 3' | | Tm °C | SB | PB | H | PIC | E | MI | DP |
| --- | --- | --- | --- | --- | --- | --- | --- | --- | --- | --- | --- |
| Me1Em1 | F1R1 | F:TGAGTCCAAACCGGTAG | R: GACTGCGTACGAATTCTG | 50 | 13 | 12 | 0.561 | 0.468 | 1.000 | 0.561 | 0.422 |
| Me1Em4 | F1R4 | F:TGAGTCCAAACCGGTAG | R: GACTGCGTACGAATTCGA | 50 | 17 | 17 | 0.550 | 0.462 | 1.000 | 0.550 | 0.402 |
| Me1Em5 | F1R5 | F:TGAGTCCAAACCGGTAG | R: GACTGCGTACGAATTCAG | 50 | 15 | 13 | 0.568 | 0.472 | 1.000 | 0.568 | 0.441 |
| Me1Em6 | F1R6 | F:TGAGTCCAAACCGGTAG | R: GACTGCGTACGAATTTGA | 50 | 13 | 10 | 0.524 | 0.446 | 1.000 | 0.524 | 0.426 |
| Me1Em7 | F1R7 | F:TGAGTCCAAACCGGTAG | R: GACTGCGTACGAATTGCA | 50 | 18 | 17 | 0.564 | 0.470 | 1.000 | 0.564 | 0.442 |
| Me1Em11 | F1R11 | F:TGAGTCCAAACCGGTAG | R: GACTGCGTACGAATTGAC | 50 | 8 | 5 | 0.563 | 0.470 | 1.000 | 0.563 | 0.492 |
| Me2Em2 | F2R2 | F: TGAGTCCAAACCGGTCC | R: GACTGCGTACGAATTGTC | 50 | 6 | 6 | 0.563 | 0.469 | 1.000 | 0.563 | 0.410 |
| Me3Em3 | F3R3 | F: TGAGTCCAAACCGGTCA | R: GACTGCGTACGAATTAAT | 50 | 14 | 14 | 0.563 | 0.469 | 1.000 | 0.563 | 0.385 |
| Me3Em4 | F3R4 | F: TGAGTCCAAACCGGTCA | R: GACTGCGTACGAATTCGA | 50 | 13 | 9 | 0.540 | 0.455 | 1.000 | 0.540 | 0.456 |
| Me4Em8 | F4R8 | F: TGAGTCCAAACCGGTTG | R: GACTGCGTACGAATTGGT | 50 | 6 | 6 | 0.565 | 0.471 | 1.000 | 0.565 | 0.446 |
| Me5Em1 | F5R1 | F: TGAGTCCAAACCGGTGC | R: GACTGCGTACGAATTCTG | 50 | 8 | 7 | 0.553 | 0.463 | 1.000 | 0.553 | 0.404 |
| Me5Em2 | F5R2 | F: TGAGTCCAAACCGGTGC | R: GACTGCGTACGAATTGTC | 50 | 16 | 15 | 0.546 | 0.459 | 1.000 | 0.546 | 0.419 |
| Me5Em3 | F5R3 | F: TGAGTCCAAACCGGTGC | R: GACTGCGTACGAATTAAT | 50 | 12 | 11 | 0.532 | 0.450 | 1.000 | 0.532 | 0.432 |
| Me5Em4 | F5R4 | F: TGAGTCCAAACCGGTGC | R: GACTGCGTACGAATTCGA | 50 | 10 | 9 | 0.567 | 0.472 | 1.000 | 0.567 | 0.388 |
| Me5Em9 | F5R9 | F: TGAGTCCAAACCGGTGC | R: GACTGCGTACGAATTCAA | 50 | 11 | 11 | 0.568 | 0.472 | 1.000 | 0.568 | 0.431 |
| Me5Em10 | F5R10 | F: TGAGTCCAAACCGGTGC | R: GACTGCGTACGAATTTGC | 50 | 3 | 0 | 0.142 | 0.132 | 1.000 | 0.142 | 0.000 |
| Me6Em1 | F6R1 | F: TGAGTCCAAACCGGACC | R: GACTGCGTACGAATTCTG | 50 | 6 | 6 | 0.535 | 0.452 | 1.000 | 0.535 | 0.338 |
| Me6Em2 | F6R2 | F: TGAGTCCAAACCGGACC | R: GACTGCGTACGAATTGTC | 50 | 11 | 9 | 0.567 | 0.472 | 1.000 | 0.567 | 0.453 |
| Me6Em3 | F6R3 | F: TGAGTCCAAACCGGACC | R: GACTGCGTACGAATTAAT | 50 | 7 | 2 | 0.429 | 0.381 | 1.000 | 0.429 | 0.344 |
| Me6Em4 | F6R4 | F: TGAGTCCAAACCGGACC | R: GACTGCGTACGAATTCGA | 50 | 9 | 8 | 0.564 | 0.470 | 1.000 | 0.564 | 0.504 |
| Me6Em5 | F6R5 | F: TGAGTCCAAACCGGACC | R: GACTGCGTACGAATTCAG | 50 | 7 | 6 | 0.417 | 0.372 | 1.000 | 0.417 | 0.271 |
| Me6Em12 | F6R12 | F: TGAGTCCAAACCGGACC | R: GACTGCGTACGAATTTAG | 50 | 9 | 9 | 0.568 | 0.472 | 1.000 | 0.568 | 0.419 |
| Total |  |  |  |  | 232 | 202 | -- | -- | -- | -- | -- |
| Average |  |  |  |  | 10.545 | 9.200 | 0.525 | 0.442 | 1.000 | 0.525 | 0.397 |

F (forward), R (reverse), Tm (annealing temperature), SB (scored bands), PB (polymorphic bands), H (expected heterozygosity), PIC (polymorphism information content), E or EMR (Effective multiplex ratio), MI (marker index) and DP (discriminating power)

**Table S5** Efficacy of 25 TRAP primers polymorphism calculated with iMEC of barley genotypes.

|  | TRAP | Sequences from 5' to 3' | | Tm °C | | SB | | PB | H | | PIC | | E | | MI | | DP | |  |
| --- | --- | --- | --- | --- | --- | --- | --- | --- | --- | --- | --- | --- | --- | --- | --- | --- | --- | --- | --- |
| T1 | F1R1 | F: TGAGTCCAAACCGGTAG | R: TCACCCGCACCTTCTTCC | 50 | | 5 | | 1 | 0.223 | | 0.211 | | 1.000 | | 0.223 | | 0.092 | |  |
| T2 | F1R2 | F: TGAGTCCAAACCGGTAG | R: CGGACAGTGGCGGAGTTA | 50 | | 8 | | 6 | 0.565 | | 0.471 | | 1.000 | | 0.565 | | 0.505 | |  |
| T3 | F1R3 | F: TGAGTCCAAACCGGTAG | R: GGCGAACTCCGACATCTT | 50 | | 5 | | 0 | 0.142 | | 0.132 | | 1.000 | | 0.142 | | 0.000 | |  |
| T4 | F1R4 | F: TGAGTCCAAACCGGTAG | R: GAGGAAGACGACGAGGAGT | 50 | | 2 | | 0 | 0.142 | | 0.132 | | 1.000 | | 0.142 | | 0.000 | |  |
| T5 | F1R5 | F: TGAGTCCAAACCGGTAG | R: CAGGCAAGACGCAAGGTG | 50 | | 5 | | 3 | 0.566 | | 0.471 | | 1.000 | | 0.566 | | 0.508 | |  |
| T6 | F1R7 | F: TGAGTCCAAACCGGTAG | R: TCCTACAAACATTGCCTACT | 50 | | 4 | | 1 | 0.242 | | 0.228 | | 1.000 | | 0.242 | | 0.115 | |  |
| T7 | F2R1 | F: TGAGTCCAAACCGGTCC | R: TCACCCGCACCTTCTTCC | 50 | | 10 | | 4 | 0.541 | | 0.456 | | 1.000 | | 0.541 | | 0.453 | |  |
| T8 | F2R3 | F: TGAGTCCAAACCGGTCC | R: GGCGAACTCCGACATCTT | 50 | | 2 | | 1 | 0.272 | | 0.255 | | 1.000 | | 0.272 | | 0.154 | |  |
| T9 | F2R5 | F: TGAGTCCAAACCGGTCC | R: CAGGCAAGACGCAAGGTG | 50 | | 5 | | 2 | 0.318 | | 0.294 | | 1.000 | | 0.318 | | 0.215 | |  |
| T10 | F3R5 | F: TGAGTCCAAACCGGTCA | R: CAGGCAAGACGCAAGGTG | 50 | | 11 | | 6 | 0.486 | | 0.421 | | 1.000 | | 0.486 | | 0.380 | |  |
| T11 | F5R8 | F: TGAGTCCAAACCGGTGC | R: TCACCCGCACCTTCTTCC | 50 | | 14 | | 9 | 0.485 | | 0.420 | | 1.000 | | 0.485 | | 0.391 | |  |
| T12 | F9R2 | F: TGAGTCCAAACCGGAGC | R: CGGACAGTGGCGGAGTTA | 50 | | 8 | | 5 | 0.541 | | 0.456 | | 1.000 | | 0.541 | | 0.423 | |  |
| T13 | F9R3 | F: TGAGTCCAAACCGGAGC | R: GGCGAACTCCGACATCTT | 50 | | 10 | | 6 | 0.494 | | 0.426 | | 1.000 | | 0.494 | | 0.409 | |  |
| T14 | F9R4 | F: TGAGTCCAAACCGGAGC | R: GAGGAAGACGACGAGGAGT | 50 | | 6 | | 2 | 0.328 | | 0.303 | | 1.000 | | 0.328 | | 0.221 | |  |
| T15 | F9R5 | F: TGAGTCCAAACCGGAGC | R: CAGGCAAGACGCAAGGTG | 50 | | 6 | | 3 | 0.423 | | 0.377 | | 1.000 | | 0.423 | | 0.354 | |  |
| T16 | F10R2 | F: TGAGTCCAAACCGGAAT | R: CGGACAGTGGCGGAGTTA | | 50 | | 9 | 5 | | 0.535 | | 0.452 | | 1.000 | | 0.535 | | 0.453 | |
| T17 | F10R3 | F: TGAGTCCAAACCGGAAT | R: GGCGAACTCCGACATCTT | 50 | | 10 | | 5 | 0.431 | | 0.383 | | 1.000 | | 0.431 | | 0.338 | |  |
| T18 | F10R4 | F: TGAGTCCAAACCGGAAT | R: GAGGAAGACGACGAGGAGT | 50 | | 9 | | 5 | 0.491 | | 0.424 | | 1.000 | | 0.491 | | 0.397 | |  |
| T19 | F10R5 | F: TGAGTCCAAACCGGAAT | R: CAGGCAAGACGCAAGGTG | 50 | | 8 | | 6 | 0.561 | | 0.468 | | 1.000 | | 0.561 | | 0.489 | |  |
| T20 | F10R6 | F: TGAGTCCAAACCGGAAT | R: CCCTCCACCAATCACAAT | 50 | | 10 | | 7 | 0.521 | | 0.443 | | 1.000 | | 0.521 | | 0.434 | |  |
| T21 | F10R7 | F: TGAGTCCAAACCGGAAT | R: TCCTACAAACATTGCCTACT | 50 | | 6 | | 4 | 0.504 | | 0.432 | | 1.000 | | 0.504 | | 0.462 | |  |
| T22 | F10R8 | F: TGAGTCCAAACCGGAAT | R: TCACCCGCACCTTCTTCC | 50 | | 9 | | 7 | 0.567 | | 0.472 | | 1.000 | | 0.567 | | 0.491 | |  |
| T23 | F11R5 | F: TGAGTCCAAACCGGTAA | R: CAGGCAAGACGCAAGGTG | 50 | | 6 | | 5 | 0.568 | | 0.472 | | 1.000 | | 0.568 | | 0.446 | |  |
| T24 | F13R1 | F: TGCCGCTTCCAACACCAA | R: TCACCCGCACCTTCTTCC | 50 | | 6 | | 4 | 0.494 | | 0.426 | | 1.000 | | 0.494 | | 0.446 | |  |
| T25 | F14R9 | F: AGTAACCCACCGCCTCCTTC | R: TTCTTCCTCCCGCTCATCCT | 50 | | 6 | | 3 | 0.547 | | 0.460 | | 1.000 | | 0.547 | | 0.482 | |  |
|  | Total |  |  |  | | 180 | | 100 | -- | | -- | | -- | | -- | | -- | |  |
|  | Average |  |  |  | | 7.2 | | 4 | 0.440 | | 0.379 | | 1.000 | | 0.440 | | 0.346 | |  |

F (forward), R (reverse), Tm (annealing temperature), SB (scored bands), PB (polymorphic bands), H (expected heterozygosity), PIC (polymorphism information content), E or EMR (Effective multiplex ratio), MI (marker index) and DP (discriminating power)

**Table S6** Efficacy of 30 SSR primers polymorphism calculated with iMEC of barley genotypes.

| Index | Sequences from 5' to 3' | | Tm °C | SB | PB | H | PIC | E | MI | DP |
| --- | --- | --- | --- | --- | --- | --- | --- | --- | --- | --- |
| bmac0090 | F:ACATCAACCCTCCTGCTC | R:CCGCACATAGTGGTTACATC | 58 | 2 | 1 | 0.565 | 0.471 | 1.000 | 0.565 | 0.846 |
| bmac0154 | F:CTGGGTGATGAATAGAGTTTC | R:TATTCTTCAAAAGATGTTCTGC | 58 | 2 | 2 | 0.556 | 0.465 | 1.000 | 0.556 | 0.462 |
| bmag0841 | F:GGAAAGTACTTCAAACCTGAA | R:CTTACAAGATGATGAGAACGA | 55 | 1 | 1 | 0.521 | 0.443 | 1.000 | 0.521 | 0.000 |
| bmag0877 | F:AAAGCTCATGGTAGATCAAGA | R:TAGTTTTCCCAAAAGCTTCTA | 55 | 2 | 2 | 0.568 | 0.472 | 1.000 | 0.568 | 0.923 |
| bmag0905 | F:TTTATCTCCCCCTAGATAGAAG | R:TCTCCGTATATTTAGGAAACG | 55 | 2 | 2 | 0.568 | 0.472 | 1.000 | 0.568 | 0.923 |
| bmag0014 | F:GCAGGGGTTGAACATCTCAT | R:CACAGGGAAACAGCTATGACC | 60 | 2 | 2 | 0.462 | 0.404 | 1.000 | 0.462 | 0.462 |
| bmac0658 | F:GTATGCAAGTGTAGGTGTGTG | R:CATGGGTTTACCCACATAC | 58 | 2 | 1 | 0.541 | 0.456 | 1.000 | 0.541 | 0.692 |
| bmag0808 | F:TCATAGACTACGACGAAGATG | R:TCTTTGGATGTGTGTTTACTG | 55 | 2 | 2 | 0.568 | 0.472 | 1.000 | 0.568 | 0.923 |
| HVOle | F:GATGGATGTCAGTCGGTC | R:ATGAGCAGTAGTACAACTCTAAGC | 53 | 2 | 2 | 0.541 | 0.456 | 1.000 | 0.541 | 0.692 |
| bmag0105 | F:AATCAGACCCATCAGAGGT | R:CCGGTCTCATAGAAATGG | 55 | 2 | 1 | 0.462 | 0.404 | 1.000 | 0.462 | 0.462 |
| bmag0718 | F:ATCGTGACATCTCAAGAACA | R:CCTGATACTGCCTAGCATTAG | 55 | 2 | 2 | 0.565 | 0.471 | 1.000 | 0.565 | 0.385 |
| ebmac0405 | F:ATGTAGCTCGGAATGTGTAGT | R:CATGTTGGATAAGAGTAGAGGA | 55 | 2 | 1 | 0.272 | 0.255 | 1.000 | 0.272 | 0.154 |
| bmac0144 | F:TACGTGTACATACTCTACGATTTG | R:ACTTATTCTGCATCCTGGGT | 55 | 2 | 1 | 0.541 | 0.456 | 1.000 | 0.541 | 0.692 |
| bmac0093 | F:CGTTTGGGACGTATCAAT | R:GGGAGTCTTGAGCCTACTG | 55 | 3 | 3 | 0.568 | 0.472 | 1.000 | 0.568 | 0.564 |
| bmac0576 | F:CAATTGTAGCCTAGCTGGTCG | R:GGGTGTATGCAAGTGGGC | 53 | 2 | 1 | 0.462 | 0.404 | 1.000 | 0.462 | 0.462 |
| bmag0115 | F:TGATCTGTGAGTAGTTCACCA | R:AATTGAAGATGCGATGATC | 63 | 1 | 1 | 0.272 | 0.255 | 1.000 | 0.272 | NaN |
| bmag0350 | F:AGCTAGATCTCTCTGTCTCTGTC | R:AAGAAACACACCACAAAGATT | 58 | 2 | 2 | 0.568 | 0.472 | 1.000 | 0.568 | 0.923 |
| bmag0013 | F:AAGGGGAATCAAAATGGGAG | R:TCGAATAGGTCTCCGAAGAAA | 58 | 3 | 3 | 0.521 | 0.443 | 1.000 | 0.521 | 0.615 |
| bmac0030 | F:CCCAATCGGAGTTACAGATG | R:GCCTCTCTGAGAATGGATC | 60 | 2 | 2 | 0.565 | 0.471 | 1.000 | 0.565 | 0.846 |
| bmac0096 | F:GCTATGGCGTACTATGTATGGTTG | R:TCACGATGAGGTATGATCAAAGA | 58 | 2 | 1 | 0.272 | 0.255 | 1.000 | 0.272 | 0.154 |
| bmac0284 | F:GCACAAAGTCATTACATCAAA | R:GATGCGTGAGTAGTTCACC | 58 | 2 | 2 | 0.568 | 0.472 | 1.000 | 0.568 | 0.923 |
| bmac0303 | F:CCTCCAAGATTAGATCTCTCTC | R:CCGTATATTTAAGAAATGGTGA | 58 | 2 | 2 | 0.541 | 0.456 | 1.000 | 0.541 | 0.692 |
| bmag0113 | F:GGAATCTTCTGGAACGTC | R:TTAAGAAGATCATTGTATTGAAGA | 55 | 2 | 2 | 0.494 | 0.426 | 1.000 | 0.494 | 0.538 |
| bmac0282 | F:CACACATACCACGCATGT | R:ATGTAAAATGGACGTATCACC | 55 | 1 | 0 | 0.494 | 0.426 | 1.000 | 0.494 | 0.538 |
| bmag0009 | F:AAGTGAAGCAAGCAAACAAACA | R:ATCCTTCCATATTTTGATTAGGCA | 58 | 2 | 2 | 0.565 | 0.471 | 1.000 | 0.565 | 0.846 |
| bmag0173 | F:CATTTTTGTTGGTGACGG | R:ATAATGGCGGGAGAGACA | 58 | 2 | 2 | 0.541 | 0.456 | 1.000 | 0.541 | 0.692 |
| bmag0219 | F:ATATTTATGAAACGGTGAAGC | R:GGGTTTATCCTCTGGTCC | 58 | 2 | 1 | 0.556 | 0.465 | 1.000 | 0.556 | 0.769 |
| bmac0127 | F:AACTATGTCCAGTCGTTTCC | R:CTTGTCGTATCATCTTATTCAGA | 58 | 3 | 2 | 0.567 | 0.471 | 1.000 | 0.567 | 0.564 |
| bmag0121 | F:ATAAGATAGGTCACCGCAATA | R:AGTAGTTCAATACAGACCTACAGG | 55 | 2 | 1 | 0.565 | 0.471 | 1.000 | 0.565 | 0.846 |
| bmac0297 | F:ATAGAGGGGGTGAAGAATAAC | R:AATAAGTGAATGATGTTGAGGA | 55 | 4 | 4 | 0.567 | 0.472 | 1.000 | 0.567 | 0.603 |
| Total |  |  |  | 62 | 51 | -- | -- | -- | -- | -- |
| Average |  |  |  | 2.066 | 1.7 | 0.514 | 0.435 | 1.000 | 0.514 | 0.627 |

F (forward), R (reverse), Tm (annealing temperature), SB (scored bands), PB (polymorphic bands), H (expected heterozygosity), PIC (polymorphism information content), E or EMR (Effective multiplex ratio), MI (marker index) and DP (discriminating power)

**Table S7 Similarity matrix for the twelve barley genotypes based on ISTR, SRAP, TRAP and SSR markers data.**

|  | KSU101 | KSU102 | Giza-124 | Gustoe | KSU-103 | Giza-121 | KSU104 | Giza-126 | Sahrawy | KSU105 | Asser | Giza-123 |
| --- | --- | --- | --- | --- | --- | --- | --- | --- | --- | --- | --- | --- |
| KSU101 | 1 |  |  |  |  |  |  |  |  |  |  |  |
| KSU102 | 0.64 | 1.00 |  |  |  |  |  |  |  |  |  |  |
| Giza-124 | 0.58 | 0.74 | 1.00 |  |  |  |  |  |  |  |  |  |
| Gustoe | 0.62 | 0.80 | 0.73 | 1.00 |  |  |  |  |  |  |  |  |
| KSU-103 | 0.52 | 0.57 | 0.64 | 0.58 | 1.00 |  |  |  |  |  |  |  |
| Giza-121 | 0.50 | 0.55 | 0.64 | 0.57 | 0.75 | 1.00 |  |  |  |  |  |  |
| KSU104 | 0.60 | 0.66 | 0.66 | 0.69 | 0.58 | 0.59 | 1.00 |  |  |  |  |  |
| Giza-126 | 0.63 | 0.69 | 0.69 | 0.65 | 0.60 | 0.61 | 0.75 | 1.00 |  |  |  |  |
| Sahrawy | 0.56 | 0.65 | 0.69 | 0.68 | 0.57 | 0.58 | 0.72 | 0.73 | 1.00 |  |  |  |
| KSU105 | 0.48 | 0.57 | 0.62 | 0.57 | 0.64 | 0.67 | 0.58 | 0.59 | 0.60 | 1.00 |  |  |
| Asser | 0.57 | 0.63 | 0.64 | 0.66 | 0.58 | 0.58 | 0.68 | 0.64 | 0.69 | 0.57 | 1.00 |  |
| Giza-123 | 0.59 | 0.67 | 0.72 | 0.70 | 0.61 | 0.62 | 0.70 | 0.72 | 0.75 | 0.61 | 0.72 | 1.00 |
